# A systematic review and meta-analysis of the benefits of school-based, peer-led interventions for leaders

**DOI:** 10.1038/s41598-022-25662-9

**Published:** 2022-12-08

**Authors:** Levi Wade, Angus A. Leahy, Mark J. Babic, Mark R. Beauchamp, Jordan J. Smith, Sarah G. Kennedy, James Boyer, Nicole Nathan, Katie Robinson, David R. Lubans

**Affiliations:** 1 Centre for Active Living and Learning, College of Human and Social Futures, University of Newcastle, University Drive, Callaghan, NSW 2308 Australia; 2 Active Living Research Program, Hunter Medical Research Institute, New Lambton Heights, NSW 2305 Australia; 3 School of Kinesiology, University of British Columbia, Vancouver, BC Canada; 4 School of Health Sciences, Western Sydney University, Penrith, NSW Australia; 5 New South Wales Department of Education, Sydney, NSW Australia; 6 Hunter New England Population Health, Wallsend, NSW Australia; 7 School of Medicine and Public Health, The University of Newcastle, Callaghan, NSW Australia; 8 Hunter Medical Research Institute, New Lambton Heights, NSW 2305 Australia; 9 Faculty of Sport and Health Sciences, University of Jyväskylä, Jyväskylä, Finland

**Keywords:** Human behaviour, Quality of life

## Abstract

The aim of our systematic review and meta-analysis was to quantitatively synthesise the effects of school-based peer-led interventions on leaders’ academic, psychosocial, behavioural, and physical outcomes. Eligible studies were those that: (i) evaluated a school-based peer-led intervention using an experimental or quasi-experimental study design, (ii) included an age-matched control or comparison group, and (iii) evaluated the impact of the intervention on one or more leader outcomes. Medline, Sportdiscus, Psychinfo, Embase, and Scopus online databases were searched on the 24th of October, 2022 which yielded 13,572 results, with 31 included in the narrative synthesis and 12 in the meta-analysis. We found large positive effects for leaders’ attitudes toward bullying (d = 1.02), small-to-medium positive effects for leaders’ literacy (d = 0.39), and small positive effects for leaders’ self-esteem (d = 0.18). There were mixed findings for behavioural outcomes and null effects for physical outcomes. Notable limitations of this research are the inclusion of a relatively small number of studies, and high heterogeneity in those included. Our findings have the potential to inform educational practice, but also highlight the need for further research examining the mechanisms that might account for the observed effects. Our systematic review was prospectively registered with PROSPERO (CRD42021273129).

Peer-led interventions typically involve the use of individuals who have volunteered or been selected to offer information, guidance, and/or support to their peers to achieve outcomes of interest^1,2^. Peer-led interventions have been described and conceptualised in a variety of ways in different fields of research, where the term “peer leader” is often used interchangeably with “peer mentor”, “student leader”, “peer advocate”, “peer support” and “peer tutor”^2–5^. Consistent with previous research^5^, we will use the terms “peer leader” or “leader” to describe participants who have delivered an intervention to their peers, and “peer recipient” to describe participants who have received an intervention from their peers. We will use the term “peer-led intervention” to describe all interventions and programs that have been delivered by peer leaders.

As young people spend a significant portion of their time in schools with their peers, appropriately designed school-based, peer-led interventions may have lasting benefits for peer leaders as well as the students they lead. School-based, peer-led interventions have been utilised to improve students’ academic, psychosocial, behavioural, and physical outcomes. The objectives of these interventions have varied, ranging from a focus on improving students’ mathematical ability^6^ and mental health^5^, through to a focus on improving nutrition^7^ and increasing physical activity^8^. The way student leaders deliver these interventions has also varied. Many peer-led interventions position peer leaders as lay teachers who deliver content to their peers, whilst others have required leaders to model certain behaviours, such as being physically active^8^ or drinking more water^9^. Several interventions have recruited peer leaders to disseminate information using more covert methods, including through informal conversations with friends^10,11^. Despite the differences in content and delivery, when considered as a whole, the findings suggest peer-led interventions have meaningful benefits for peer recipients.

Throughout childhood, social interactions amongst peers are replete with information sharing, whereby individuals act as both teachers and learners. Encouraging students to serve as peer leaders is a means of formalising this process, with the valuable addition of support and tutelage provided by teachers and/or research staff. Interestingly, despite the distinct roles played by students within peer-led interventions, research often focuses solely on outcomes for peer recipients. Though scant, the available evidence suggests peer leaders may also benefit to a similar, or perhaps even greater extent. For example, a recent review of peer-led mental health interventions found benefits for leaders’ self-esteem and a reduction in their stress^5^. Recent data also suggest peer-led academic interventions may improve leaders’ mathematics and reading ability^12^. However, previous reviews on the effects of such interventions for leaders’ outcomes have been limited by several important factors that undermine the generalisability of their findings. Some have included studies that utilised reciprocal teaching methods (where a student is both a leader and a student being led)^13^, thus making it difficult to parse the effects of being a peer leader versus being a peer recipient. Others have included studies where peer-teaching is one of many (potentially impactful) intervention components, limiting the ability to attribute the effects to the act of peer-leadership^8^. Finally, other reviews have included research focused on children with disabilities or behavioural issues^12^, which limits insights into how efficacious peer-led interventions might be in other (i.e., mainstream) educational settings.

Identifying the effects of school-based peer-led interventions on valued outcomes for leaders is important, given the involvement of leaders is a central element. If clear benefits for both leaders and peer recipients can be demonstrated, it would further bolster the rationale for the implementation of peer-led interventions within school systems. However, the extant literature does not yet allow for conclusions regarding the capacity of peer-led interventions to derive salient benefits for those acting as leaders themselves. To address this gap in the literature, the aim of this systematic review and meta-analysis was to evaluate the effect of school-based peer-led interventions on child and adolescent peer-leaders’ academic, psychosocial, behavioural, and physical outcomes.

## Methods

Our systematic review was prospectively registered with PROSPERO (CRD42021273129) and carried out following the Preferred Reporting Items for Systematic Reviews and Meta-Analyses (PRISMA) recommendations^14^, the PRISMA checklist is attached as Appendix A. We conducted a systematic search of four electronic databases (MEDLINE, SPORTDiscus, PsychInfo, Embase, and Scopus) on the 24th of October, 2022. The keywords used in the search were: child* or adolescen* or youth* or teen* or student or “young people” or “young person” AND RCT or “randomized controlled trial” or “randomised controlled trial” or experiment* or quasi-experiment* or intervention AND “peer-led*” or leader* or tutor* or tutee or “peer-assisted learning” or mentor* AND School* or elementary* or “high school” or “primary school” or “middle school” or education*. Medical sub-headings were applied where possible. An example of the search strategy is available in Appendix B.

Only studies published in English and in a peer-reviewed journal were included. No limitations on publication date were applied during the search. The titles and abstracts of the search results were screened by the first author against inclusion criteria. The full-text of potentially relevant studies were retrieved and reviewed by two authors (L.W and A.A.L) to determine eligibility in the review. Finally, the reference lists of all included articles and previous reviews^5,8,12,13,15^ on the topic were also checked to identify any articles that were not located via the initial search. We used Covidence systematic review software (Veritas Health Innovation, Melbourne, Australia) for title and abstract screening and full-text review.

### Criteria for inclusion/exclusion

The following eligibility criteria was applied to the retrieved studies: *Population:* participants were apparently healthy children (aged 5 to 12) or adolescents (aged 13 to 18). *Intervention:* school-based interventions (e.g., educational, experiential, health promotion) delivered by a child or adolescent student to other students. *Comparison:* included an age-matched comparison group who were not involved in the peer-led intervention. This includes waitlist controls and treatment as usual comparisons. *Outcomes:* the study included at least one academic, psychosocial, behavioural, or physical outcome as either a primary or secondary outcome. For the purposes of this review, we conceptualized:

- “Academic” outcomes as factors associated with academic performance at school, including but not limited to classroom behaviour, literacy outcomes, cognitive function, and academic achievement.
- “Psychosocial” outcomes as outcomes related to feelings, beliefs, ways of coping, and relationships with others. This includes mental health outcomes (e.g., self-esteem, anxiety) and social outcomes (e.g., bullying, victimisation).
- “Behavioural” outcomes as health-related behaviours, including physical activity, diet, and sleep.
- “Physical” outcomes as health and movement-related performance outcomes, including fitness (e.g., cardiorespiratory fitness, muscular fitness), movement skill competency, and other physiological markers of health (e.g., blood pressure and body mass index).

*Study design:* Only studies using an experimental or quasi-experimental study design (including non-randomised controlled trials) were eligible.

Excluded studies were those that: (i) included reciprocal teaching methods (whereby a student was both a leader and a peer recipient), (ii) focused on a special group, such as children or adolescents with autism, or those with emotional (e.g., depression) or behavioural issues (e.g., conduct disorders).

### Criteria for risk of bias assessment

Two authors (L.W and A.A.L) independently assessed the risk of bias of all included studies using the Cochrane Risk of Bias Tool (RoB 2.0)^16,17^. Where these authors could not agree, a third author was consulted (D.R.L), and consensus reached. Each study was rated against five criteria relating to the risk of bias due to: (i) randomisation process, (ii) deviations from intended interventions, (iii) missing outcome data, (iv) measurement of the outcomes, and (v) selection of the reported results. Domain-specific judgements of each paper (“low risk”, “some concerns”, or “high risk” of bias) were made after rating each criterion on a 4-point scale (“not applicable”, “yes / probably yes”, “no / probably no”, and “no information”). The risk of bias is included as Appendix C.

### Meta-analyses

Baseline and post-test means as well as standard deviations were used to calculate the difference between groups over time^18^. If information was not available within the published paper and/or authors failed to provide this information upon request, post-test mean values and their standard deviation were used. Cohen’s *d* along with 95% CIs and *p* values were used to determine the pooled effect of peer-led interventions. Effect sizes were interpreted as small (*d* = 0.2 to < 0.5), medium (*d* = 0.5 to < 0.8), or large (*d* ≥ 0.8)^19^. Effect sizes were combined using a multilevel meta-analysis. This approach is not limited by the assumption of independence, allowing multiple effect sizes to be calculated from each study^20^. Unconditional mixed-effects models using maximum likelihood estimation were conducted to calculate the overall pooled effect size. A 95% confidence interval was calculated for each pooled effect size. All analyses were run using the metafor package^21^ in R Version 4.0.2^22^.

Statistical heterogeneity was determined using *I*^*2*^ values. *I*^*2*^ provides an indication of what proportion of the observed variance would remain if we could eliminate the sampling error^23^. Values of < 40%, 40–75% and > 75% were interpreted as limited, substantial, and considerable heterogeneity, respectively^24^. Publication bias was assessed using funnel plots and Egger’s regression asymmetry tests^24^. Separate meta-analyses were conducted for outcomes within the academic and psychosocial categories. Due to a lack of sufficient data, the corresponding meta-analyses were not conducted for behavioural and physical outcomes. The following potential categorical moderators of effects were identified after PROSPERO registration: (i) age (child [5–12] vs. adolescent [13–18]), (ii) study design (experimental vs. quasi-experimental), (iii) study duration (≤ 10 weeks vs.  > 10 weeks) and (iv) congruence of age between leaders and peer recipients (same age vs. cross-age). Due to the homogeneity of moderators across the studies included in the meta-analyses (e.g., all studies in the meta-analyses had child leaders), we were not able to conduct moderator analyses.

### Data extraction

The following data were extracted into a customised Excel spreadsheet: author name, year of publication, country, aim of the study, sample size, age, description of the intervention and comparisons, study design, measure/s used, use of theory in the design of the intervention, whether an adjustment for clustering was used, the method of selecting leaders, and the means and standard deviations of outcomes of interest. The data were extracted by one author (L.W) and checked by another (A.A.L).

## Results

**Figure 1:**
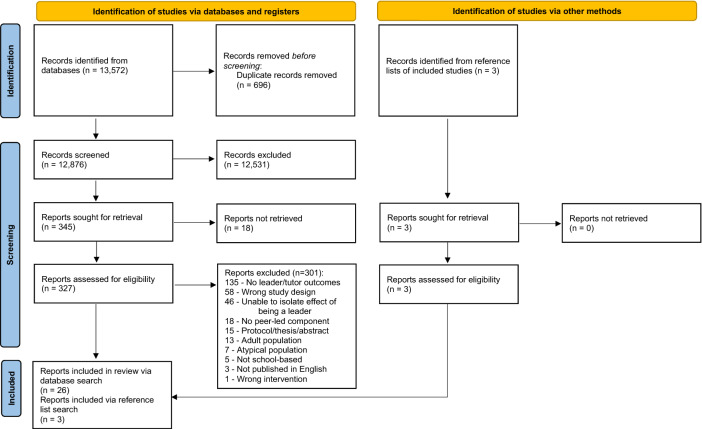
Flow of studies through the systematic review process.

The search strategy initially produced 13,572 results. After the removal of duplicates, 12,876 reports were screened by title and abstract. Of these, 29 were included in this study (see Fig. 1). One report^25^ includes data on three experiments, thus a total of 31 studies are included in this review. Of the included studies, 12 were included in the meta-analyses. The results of the meta-analyses and a narrative review of the included studies are provided in the following sections. An overview of included studies is presented in Table 1. Overall, the included studies encompass 9062 school-aged children and adolescents, with interventions most often implemented in the USA (*n* = 10) and the UK (*n* = 5). Thirteen studies used a randomised controlled trial design, and 18 used a quasi-experimental design. Thirteen of the included studies accounted for clustering effects in their analyses. The majority of the included studies were published between 2010 and 2022 (*n* = 24). Most were conducted in primary schools (*n* = 21), where the peer leaders were children (5 to 12 years old). The remaining ten studies were conducted in secondary schools with adolescent leaders (13 to 18 years old). Thirteen (42%) of the included studies cited the use of an established theory, which were most commonly Sociocultural Theory (*n* = 6)^26^, Social Cognitive Theory (*n* = 2)^27,28^, or Self-Determination Theory (*n* = 2)^29^.

**Table 1:** Summary of the included studies.

Author (year)	Leaders and Control	Mean age	Type of intervention	Theory	Intervention description	Outcomes of interest	Findings
Boulton (2016)	Int leaders: 100 Age-matched control: 46	Included sample: 11.5 years	Cross-age: grade 6 leaders, grade 4 students	Role theory, cognitive theory, sociocultural theory	Leaders worked in groups to design a 30-min lesson on online risks and how to avoid them. Leaders delivered their lesson to a group of approximately five younger students	Knowledge of online risks Knowledge of online safety	There were significant improvements in both outcomes for the leaders. There were no significant changes in the control group
Boulton (2021a)	Int leaders: 55 Age-matched control: 44	Average age in this year group = 11.5 years	Cross-age: grade 6 leaders, grade 4 students	Role theory, sociocultural theory	Leaders worked in groups to design a 30-min lesson on online risks and how to avoid them. Leaders delivered their lesson to a group of younger students	Beliefs about non-physical forms of bullying: Harmful exclusion Harmful verbal Acceptable exclusion Acceptable verbal	There were significant improvements favouring the intervention for all outcomes
Boulton (2021b)	Int leaders: 106 Age-matched control: 91	Average age in this year group = 11.5 years	Cross-age: grade 6 leaders, grade 4 students	Role Theory, Sociocultural Theory	Leaders worked in groups to design a 30-min lesson on online risks and how to avoid them. Leaders delivered their lesson to a group of younger students	Beliefs on getting help when one is bullied: Wanted help When to tell	There were significant improvements favouring the intervention for all outcomes
Boulton (2021c)	Int leaders: 76 Age-matched control: 47	Average age in this year group = 11.5 years	Cross-age: grade 6 leaders, grade 4 students	Role theory, sociocultural theory	Leaders worked in groups to design a 30-min lesson on online risks and how to avoid them. Leaders delivered their lesson to a group of younger students. Researchers delivered a 40-min presentation to those in the control group	Beliefs on supporting bullying victims: Victim support—emotional Victim support—address bully Vitim support—other	There were significant improvements favouring the intervention for all outcomes
Campbell (2012)	Int leaders: 519 Age-matched control: 214	Included sample: 16.6 years (sd = 0.83)	Cross-age: grades 4–7 tutoring K to grade 3	–	Leaders taught a 30-min lesson on healthy living to their partner via presentation, game or other activity. Each buddy pair also spent two 30-min structured physical activity sessions per week in the gymnasium, which allowed both leader and their partner to participate simultaneously	Health knowledge Attitudes towards health Self-reported healthy behaviours Lifestyle habits	The leaders in the intervention showed greater increases in healthy living knowledge and attitudes towards health. There were no significant effects on healthy behaviours or lifestyle habits
Caron (2004)	Int leaders: 147 Age-matched control: 159	Int: 16.0 years Con: 15.9 years	Cross-age: ~ 16 year-old leaders leading ~ 14 year-old students	Social cognitive theory	Leaders participated in a training program integrated into their moral and religious education classes. they worked together to develop and present a lesson on sexual health or healthy relationships	Attitude toward sexual intercourse Attitude toward condom use	The peer educators were more likely to use condoms on a regular basis than those in the control group, but did not modify their attitudes towards postponing sexual intercourse
Carruth (2010)	Int leaders: 27 Age-matched control: 16	Included sample: 15 to 19 years (no mean reported)	Same age	–	Students trained as first-aid instructors and taught these skills to their peers	First-aid knowledge Preparedness for emergency situations	Findings demonstrated significant increases in preparedness for emergency situations but not first aid knowledge among those who were trained as instructors compared with the non-intervention group
Cui (2012)	Int leaders: 59 Age-matched control: 286	Included sample: ~ 12.7 years	Same age	Social cognitive theory	Peer leaders delivered four 40-min peer education lessons to their classmates over four consecutive weeks. Content was focused on food choice, physical activity and sedentary behaviour, carbonated drinks and goal setting	Total MVPA, min/day Sedentary behaviours	There were no significant differences between peer leaders and other students in time on MVPA, sedentary behaviours or computer usage at both 3 and 7 months
Foss (2022)	Int leaders: 387 Age-matched control: 352	Included sample: 16.6 years (sd = 0.83)	Unspecified	–	Leaders are taught from a sexual health curriculum to deliver sex education to their peers (1 presentation at a school per month). Leaders attend an annual retreat, and 2-h weekly meetings throughout the school year	Confidence in peer education skills Civic action Empathy Communication with parents about sex and birth control Use of contraception Comfort with own sexuality	There were significant improvements in all outcomes favouring intervention leaders over controls
Golonka (2017)	Int leaders: 20 Age-matched control: 22	Included sample: 12.2 years	Cross-age: peer recipients were in grade six. It is implied that leaders were older	–	Student leaders met twice a week for eight weeks to develop anti-drug messages to present to sixth-graders at school. At the end of the program, leaders presented their work to sixth-graders at a school assembly	Cigarette smoking Alcohol consumption Marijuana smoking	There were no significant within-group changes for the experimental leaders, however, compared to the experimental group, there was a significant increase in alcohol consumption in the control group
Jago (2021)	Int leaders: ~ 145 Age-matched control: 800	Included sample: 13.8 years	Same age	Diffusion of innovation theory, self-determination theory	‘Peer supporters’ were encouraged to informally promote physical activity among their peer group	Weekday MVPA	Slightly larger decrease in MVPA in intervention compared to control. More pronounced decline in peer-supporters compared to non-peer supporters
Mason-Jones (2013)	Int leaders: 295 Age-matched control: 433	Included sample: grade 10	Unspecified	–	Leaders were educated on sexual health (including education on HIV), and delivered a HIV prevention program to their peers	Age of sexual debut Condom use Self-esteem Decision-making Critical thinking Goal-orientation	There was a significant improvement in peer leaders’ decision-making compared to the control group. There were no other significant effects of the program
Miller (2010)	Leader cross age: 81 Leader same age: 33 Age-matched control: 92	Included sample: 10–11 years old	Cross age: grade 6 leading grade 4 students; same age: grade 6 with grade 6	–	Paired reading (either cross-age or same-age): students read together, with the leader assisting when needed	Self-esteem Self-competence Self-worth	Significant improvements in self-esteem for both paired reading groups, improvements in self-worth in the cross-age condition
Mitchell (2016)	Trained leaders: 17 Untrained leaders: 12 Age-matched control: 14	Included sample: no age provided	Cross age: grade 4 leading grade 2	–	During each leading session, leaders assisted others to identify misspelled words in their writing and to use spelling strategies they had been taught during the lessons to fix spelling errors	Dictated writing Free writing	Despite significant improvements in dictated and free writing for the grade 2 students, there were no significant improvements in either outcome for the leaders
Nathan (2017)	Int leaders: 20 Age-matched control: 30	Included sample: 11 years	Cross age, Grade 6 (11–12 Y.O—trained) leading kindergarten to grade 2 (mean age 6.1)	Transformational leadership theory	Peer leaders led Kindergarten students through 30-min lessons focused on developing fundamental movement skills	Teacher-rated leadership Students’ perceived leadership self-efficacy	Significant improvements in teacher-rated leadership ability of leaders favouring intervention over control
Palladino (2012)	Int leaders: 42 Age-matched control: 144	Included sample: grades 9–13	Same age	–	Online cyberbullying intervention: student leaders moderated site content and delivered face-to-face education to peers	Bullying and victimisation Cyberbullying and cybervictimisation Ability to cope with stress	Significant decrease in bullying, victimisation, and cybervictimisation favouring the experimental over the control group. No differences between leaders and peer recipients
Paquette (2009)	Int leaders: 25 Age-matched control: 25	Included sample: 9–10 years	Cross age: Grade 4 (aged 9–10) leading Grade 2 (aged 7–8)	–	Cross-age writing program. Leaders and their partner discussed writing samples using a writing rubric for guidance	Writing ability	No differences in the writing ability of peer recipients. Significant improvements in writing ability of leaders when compared to control
Robinson (2007)	Int leaders: 12 Age-matched control: 11	Included sample: 10 to 11 years	Cross age: grade 5 (aged 10–11) leading kindergarten (aged 5—6)	–	Fifth Grade students were trained in filial therapy and participated in weekly play sessions with kindergarten students	Empathy	Significant improvements in empathy in experimental leaders compared to control leaders
Santos (2014)	Int: 340 Con: 307	Included sample: ~ 9 years	Cross-age: Grade 4–6 leading kindergarten to grade 3	–	Each week, the older students received a 45-min healthy living lesson from their classroom teacher. Later that week, the older students taught a 30-min lesson to their younger “buddies.” The pairs also engaged in two 30-min structured aerobic fitness sessions per week	Self-esteem Healthy habits BMI Physical activity Cardiorespiratory fitness	Despite some improvements in the peer recipients in the intervention group, there was a significant decrease in physical activity in the peer leaders. There were no other significant effects for the older students in the intervention
Sheppard (2012)	Int leaders: 22 Age-matched control: 22	Included sample: 12.2 years	Cross age: grade 7 leading grade 6	–	Leaders created and presented anti-drug messages to their peers to facilitate discussions	Leadership self-perception	There was a significant increase in female leaders’ perception of how ‘cool’ (a subscale of leadership self-perception) they are compared to control leaders
Silverman a (2017)	Int leaders: 131 Age-matched control: 106	Included sample: grade 4	Cross age: grade 4 leading kindergarten	Sociocultural theory	Leaders led another student through vocabulary, writing, and drawing activities	Vocabulary and comprehension Receptive knowledge of words Expressive knowledge of words Reading comprehension and strategy use	Peer recipients improved their vocabulary compared to control. Leaders in the intervention group improved their vocabulary, text comprehension, and strategy use compared to control
Silverman b (2017)	Int leaders: 265 Age-matched control: 214	Included sample: grade 4	Cross age: grade 4 paired with kindergarten	Sociocultural Theory	Leaders helped another student to answer questions and play games related to a video they watched. Each session, the leader would read a book aloud to their partner	Target word knowledge Reading comprehension Literacy skills	Peer recipients in the intervention group saw small improvements in target word knowledge, reading comprehension, and literacy skills. leaders improved on their target word knowledge compared to control
Smit (2016)	Int leaders: 25 Age-matched control: 104	Included sample: 10.7 years (sd = 0.78)	Same age	Self-persuasion theory, self-determination theory	Influence agents (essentially covert leaders) were taught the benefits of water consumption, to consume more water, and how to promote water consumption in their friend networks	Water consumption Sugar-sweetened beverage consumption Water-drinking intentions	There was a significant increase in water consumption and a significant decrease in sugar-sweetened beverage consumption favouring the intervention over the control group
Song (2018)	Int leaders: 121 Age-matched control: 216	Included sample: grades 7 and 8	Same age	–	Top-performing students were matched with lower-performing students and were offered incentives to study together	Mathematics ability Mental health	There was a decrease in the mental health scores of the peer recipients. There was an increase in mathematical ability and a decrease in social stress (subscale of mental health) for the leaders
Stock (2007)	Int leaders: 128 Age-matched control: 71	Included sample: no age provided	Cross age: fourth to seventh grade leaders paired with kindergarten to third grade students	–	Leaders taught a 30-min lesson on healthy living to their partner via presentation, game or other activity. Each buddy pair also spent two 30-min structured physical activity sessions per week in the gymnasium, which allowed both leader and their partner to participate simultaneously	Body image Blood pressure Cardiorespiratory fitness Health knowledge and behaviour	Peer recipients improved their health knowledge attitude towards health to a significantly greater extent than the control group. Leaders significantly improved their health knowledge, attitudes, and health behaviours compared to control
Tarro (2019)	Int leaders: 94 Age-matched control: 98	Int = 13.2 years (sd = 0.58) Con = 13.1 years (sd = 0.61)	Cross age: leaders: 12–14, peer recipients: 9–11	–	Leaders received education on nutrition, healthy lifestyle, and communication techniques. Leaders at each school designed an activity related to leading a healthy life. The leaders from each school met up and taught one another about their topic. The leaders then delivered the activities to younger students	Percentage of children practicing ≥ 6 h/week of moderate to vigorous physical activity Fruit consumption Screen-time Sugary drink consumption Fast food consumption	There were no significant changes in any of the outcomes of interest
Topping (2004)	Int leaders: 10 Age-matched control: 10	Included sample: 8 to 9 years	Cross age: leaders: 8–9, peer recipients: 7–8	–	Leaders received training on how to work with a partner. They were then paired with another student. The pair worked together on science activities for two 30-min sessions per week for eight weeks	Knowledge of words related to science	There were significant improvements for the peer recipients, but not for the leaders
Van Keer (2010)	Int leaders: 277 Age-matched control: 80	Included sample: no age provided	Cross-age: grade 6 leaders paired with grade 3 students	–	Leaders instructed another student on the use of reading strategies and read together in paired sessions	Reading strategy awareness Reading strategy use Reading comprehension and achievement	Significant effect of the intervention on the reading strategy awareness and reading strategy use of peer recipients. There were also significant effects favouring the intervention for leaders’ reading strategy use
Wong (2012)	Int leaders: 50 Age-matched control: 130	Included sample: ‘secondary grade 4'	Same age	–	The intervention group participated in a leadership program which involved participation in volunteer services and school-based moral educational programs	Self-esteem Self-efficacy	Significant improvements in both outcomes for the intervention group, but only for females
Wyman (2010)	Int leaders: 268 Age-matched control: 185	Included sample: 15.7 years (sd = 1.17)	Same age	Diffusion of innovations theory, valente’s social network thresholds model	Peer leaders were trained to disseminate messages on suicide prevention in their school	Suicide perception and norms Social connectedness Peer leader behaviours (supporting peers and referring distressed peers)	Compared to control, there was a positive effect of the intervention on peer leaders’ suicide perception and norms, social connectedness, and peer leadership behaviours
Yogev (1982)	Int leaders: 73 Age-matched control: 98	Included sample: 16 years	Cross age: 16 year-old students leading 13–14 year old students	Role-taking theory	The peer-led program is offered as an elective subject. Leaders met with another student twice a week. The students participated in role-playing, modelling, and case analyses, using closed-circuit television and other audio-visual aids as a means for analysing the role of leader	Empathy Altruism Anti-utilitarianism Self-esteem	The results of this study indicate that cross-age tutoring significantly increases the leaders' empathy, altruism, and self-esteem

Fifteen studies were delivered for ≤ 10 weeks, and 14 were implemented over > 10 weeks (we were unable to determine the length of the remaining two studies). Most of the studies utilised a cross-age (i.e., older leaders and younger peer recipients) approach (*n* = 20). The remaining 10 studies utilised same-aged peer leaders (i.e., leader and peer/s were the same age), one study compared both methods, and one did not report on this. Regarding the selection of leaders, 12 studies used random selection (via class randomisation), seven studies used a self-nomination process, four selected leaders based on their ability, three used a peer nomination system, three studies had teachers select leaders, and two studies did not report this information. All of the peer-led programs were delivered face-to-face, with one study utilising a blended format with online and face-to-face delivery.

### Risk of bias

The use of self-report outcome measures and quasi-experimental designs were the most common sources of bias. Specifically, 18/31 studies did not allocate students randomly between conditions. Of the included studies, five were considered to be at ‘high risk’ of bias due to deviations to from the intended interventions. The majority of studies were at low risk of bias attributable to missing outcome data (18/31). Most studies were at high risk of bias in measurement of the outcome (19/31). This was largely attributable to the use of self-report measures in combination with students being aware of which arm they were allocated to. No studies had a high risk of bias attributed to selection of the reported result. The risk of bias assessment is in Appendix C.

### Academic outcomes

#### Meta-analysis—literacy outcomes

**Figure 2:**
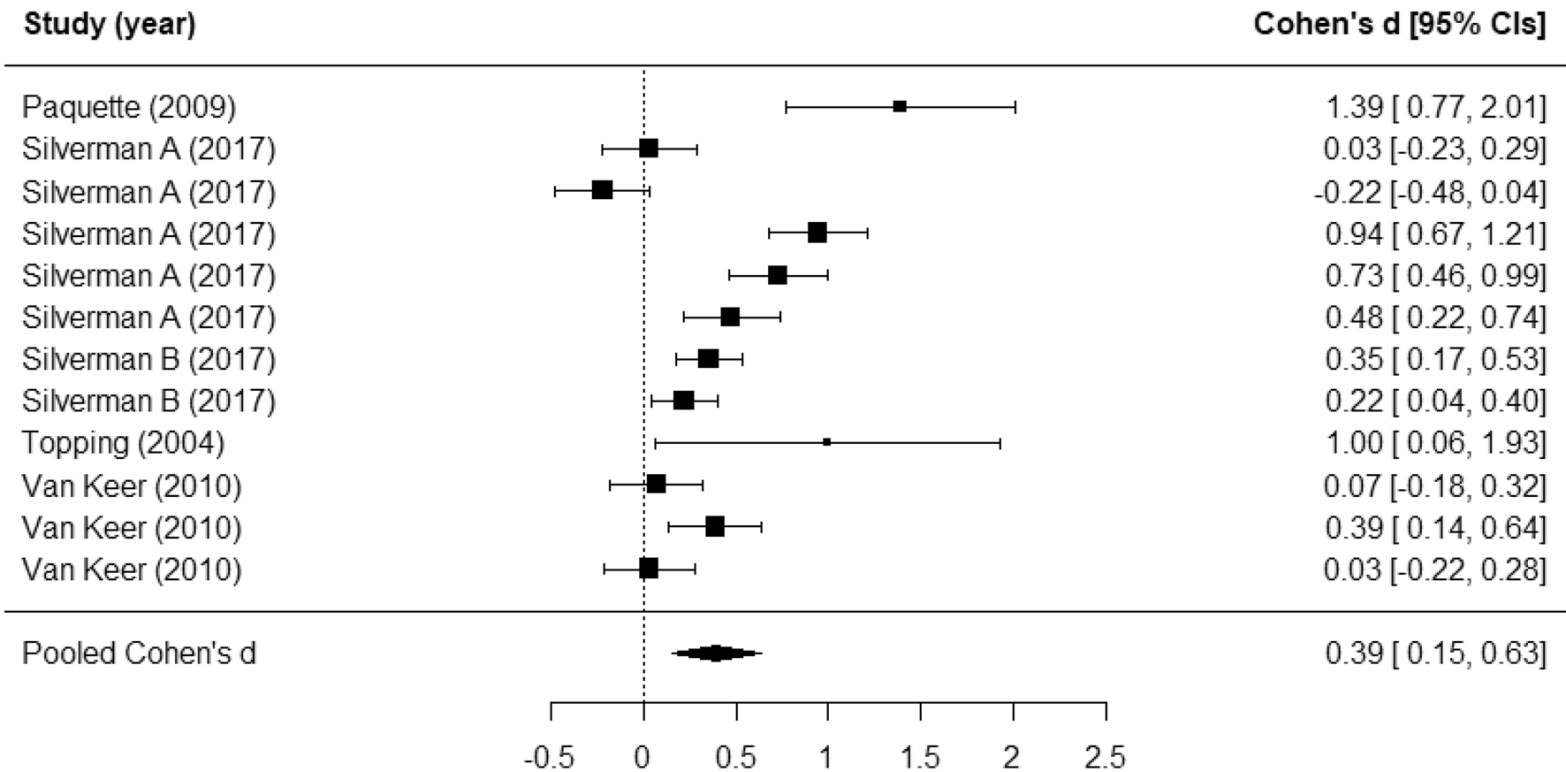
Forest plot for literacy outcomes.

Five studies reporting on literacy outcomes (including writing ability, vocabulary, and reading comprehension) were included in a meta-analysis. The meta-analysis included a total of 1143 participants from five studies (see Table 2). We found a medium-sized pooled effect on leaders’ literacy outcomes (Cohen’s *d* = 0.39, 95% CI 0.15 to 0.63; *p* = 0.001) (see Fig. 2). A considerable proportion of variation in the pooled effect was attributable to differences within studies (I^2^ = 89.71), and a limited proportion to differences between studies (I^2^ = 0.00). Egger’s test indicates that there is evidence of publication bias (*p* = 0.02).

**Table 2:** Primary meta-analyses of the effect of peer-led interventions on leaders’ outcomes.

Variable	*k*	Number of effect sizes	*d*	Lower 95% CI	Upper 95% CI	${I}^{2}\_2$	${I}^{2}\_3$	Overall *I*^*2*^	$\tau \_2$	$\tau \_3$
Literacy	5	12	0.39	0.15	0.63	0.90	0.00	0.90	0.15	0.00
Attitudes toward bullying	3	9	1.02	0.46	1.57	0.02	0.85	0.86	0.01	0.23
Self-esteem	4	5	0.18	0.07	0.30	0.00	0.00	0.00	0.00	0.00

#### Narrative synthesis

Among the eleven included studies reporting academic outcomes for leaders, ten were quasi-experimental with a control group, and one was a randomised controlled trial. The sample size of the included studies ranged from 20 to 733. The majority of interventions ran for ten weeks or less (6/11 studies). Most interventions had peer-leaders meet with peer recipients at least once per week (9/11 studies). The duration of the sessions lasted between 25 and 60 min. In most instances (9/11 studies), children (aged 5–12) were the peer-leaders. Most interventions recruited older leaders to lead a younger student or students (9/11). It was most common for leaders to be paired with another younger student (9/11), though two studies required leaders to teach to a small group of students. The majority of studies did not report the use of a particular theory in the development of their intervention (8/11).

Five studies focused on leaders’ literacy outcomes, including reading comprehension, writing ability, and vocabulary^30–34^. Four of these paired a leader with a younger peer, and required the leader to assist with reading or vocabulary tasks^30–32,34^, whereas Topping^33^ had the pairs work through science activities. Each of these studies reported improvements for the leaders. Two of these studies reported the effects of their peer-led writing interventions on leaders’ own writing skills^30,35^. Both paired students, one older and one younger, and required the older student to lead the other through certain writing tasks. The intervention described by Mitchell, et al.^35^ had the older leader help the younger student with their spelling, whilst the pairing in the intervention described by Paquette^30^ focused on discussing the qualities of writing samples (e.g., word choice, sentence fluency). Paquette^30^ reported significant improvements in the writing ability of leaders, whereas Mitchell, et al.^35^ found no significant effects on leaders’ outcomes. Two studies reported significant effects of the Healthy Buddies program on leaders’ knowledge of and attitudes towards healthy behaviours (including healthy eating and physical activity)^36,37^.The remaining studies reported on other subject-specific outcomes, finding significant positive effects on leaders’ knowledge of first-aid^38^, online safety^39^, and improvements in math ability^40^.

### Psychosocial outcomes

#### Meta-analysis—attitudes toward bullying

**Figure 3:**
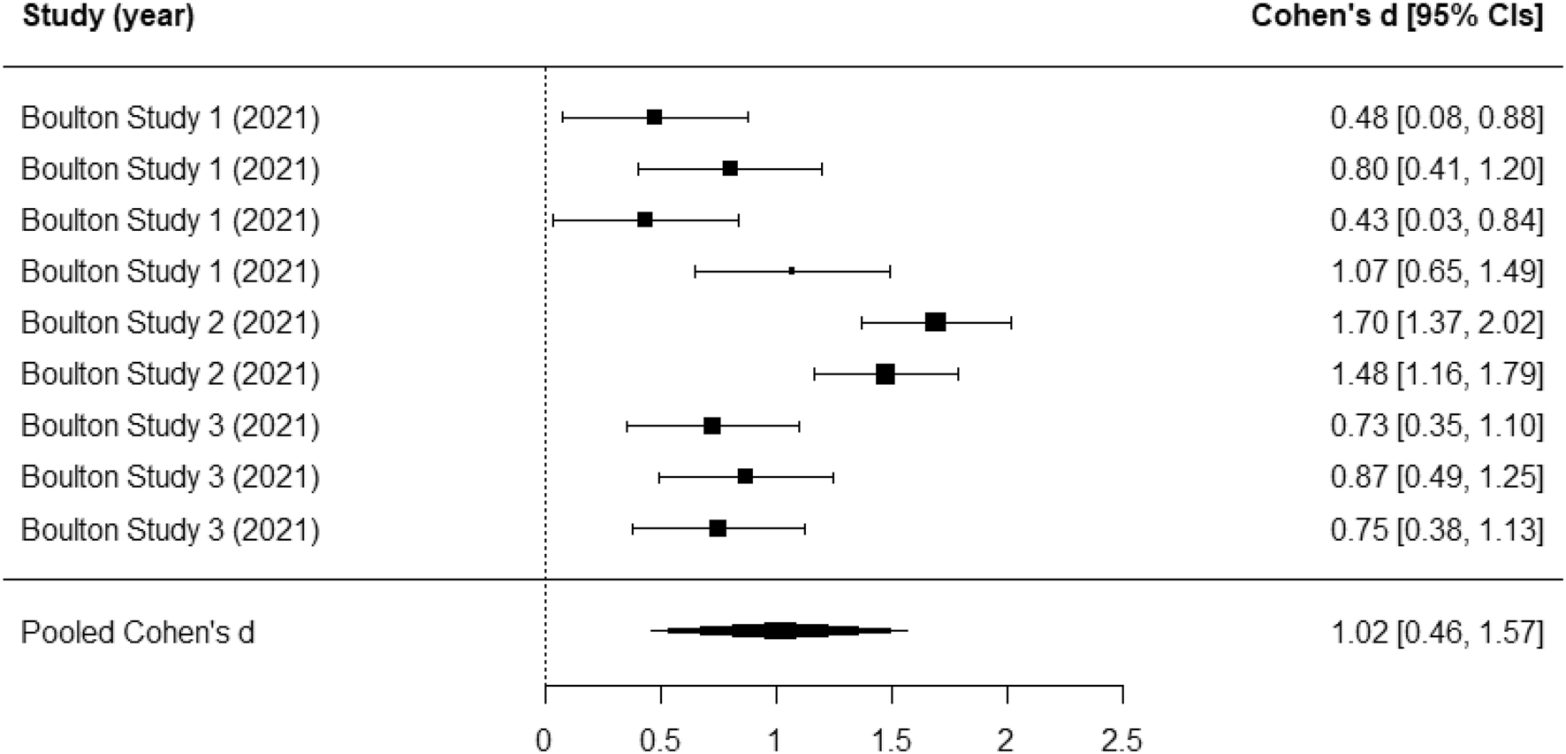
Forest plot for attitudes toward bullying.

Three studies were reported in the one paper by Boulton, et al.^25^. Each reported on attitudes towards bullying and were included in a meta-analysis. We found a large pooled effect (Cohens’ *d* = 1.02; 95% CI 0.46 to 1.57; *p* < 0.001) (see Fig. 3). A limited proportion of variation in the pooled effect was attributable to differences within studies (I^2^ = 0.02), with a considerable proportion attributable to differences between studies (I^2^ = 0.85). There was evidence of publication bias (*p* < 0.01).

#### Meta-analysis—self-esteem

**Figure 4:**
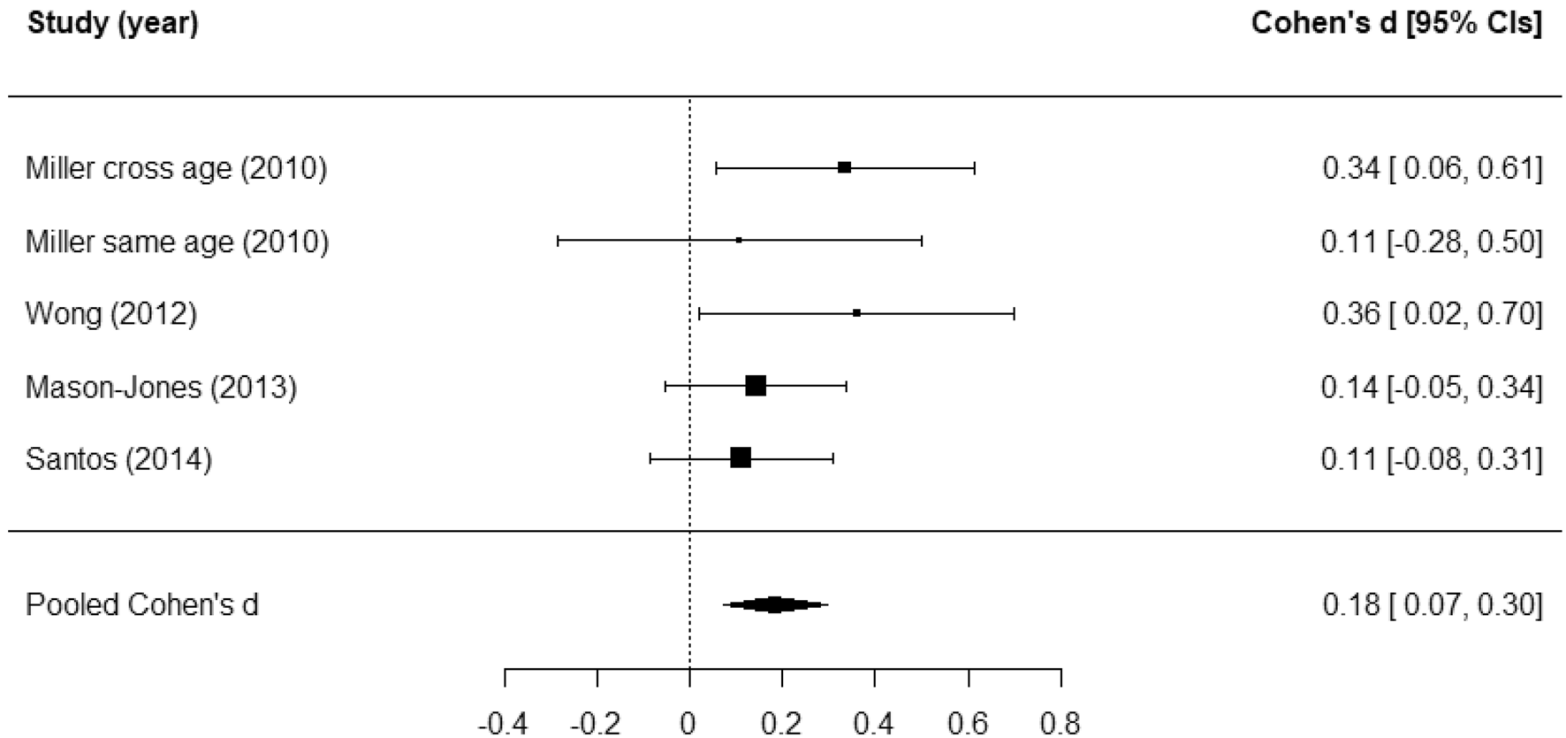
Forest plot for self-esteem.

Four studies reporting on the effects of their program on self-esteem were meta-analysed. There was a pooled effect of *d* = 0.18; 95% CI 0.07 to 0.30; *p* < 0.001) (See Fig. 4). A limited proportion of variation in the pooled effect was attributed to differences within or between studies (both I^2^ < 0.001). There was no evidence of publication bias (*p* = 0.35).

#### Narrative synthesis

This section provides further description of the 16 studies which tested peer-leaders’ psychosocial outcomes, including the seven studies included in the meta-analyses. The studies reporting on psychosocial outcomes of their peer-led intervention included eight quasi-experimental with control group and eight randomised controlled trials. The sample size of the included studies ranged from 23 to 739. Although the duration of two interventions could not be determined, most ran for more than ten weeks (8/14). Not all studies reported the number of peer-led sessions per week, though the available information indicates that in all cases, there was at least one peer-led session per week, with the majority of these meeting 2 or more times per week (8/9). The duration of these sessions lasted between 30 and 60 min. Most studies were conducted in primary schools, thus typically having child (10/15) rather than adolescent (6/15) peer-leaders. One study did not report this information. The majority of studies used cross-age methods, whereby an older leader taught a younger peer or peers (9/14) (two studies did not report this information). Whether leaders engaged in pairs or in some other manner was inconsistently reported (four studies did not provide this information), though the available information indicates that leaders often led a single student (5/11 studies) or led a group of students (6/11 studies). Regarding the use of theory, only six of the included studies cited the use of a particular theory in the development of their intervention.

Five studies examined the effect of their peer-led intervention on leaders’ self-esteem^41–45^, and another examined leaders’ body-image^37^. These interventions varied substantially in their content (i.e., sexual health, reading, healthy eating, leadership, and tutoring), as well as how they were implemented (i.e., paired and group-based interventions). Three noted statistically significant improvements in leaders’ self-esteem^41–43^, whilst Mason-Jones et al.^44^ and Santos et al.^45^ found no effect. Further, Stock et al.^37^, reported null findings for leaders’ body image.

Two studies reported on the effects of their interventions on students’ leadership abilities. Both studies had small samples and required peer-leaders to lead a group of other students. Nathan and colleagues’^46^ intervention involved older students teaching fundamental movement skills to small groups of younger students, and the authors reported improvements in peer-leaders’ leadership skills (as rated by their teachers). Alternatively, Sheppard and colleagues’^47^ peer-led drug education intervention had no effect on leaders’ self-rated leadership ability, (except for a significant effect on how “cool” they perceived themselves—a subscale of the measure of leadership self-perception).

Three studies examined whether their intervention improved leaders’ empathy. Each of these studies noted a significant positive effect on leaders’ empathy^43,48^, including a significant increase in leaders’ sense of social connectedness and ability to support distressed peers^49^. Song, et al.^40^ paired high-performing students (leaders) with same-aged lower-performing classmates and provided incentives for them to study together. They found that leaders reported significant reductions in social stress^40^.

### Behavioural outcomes

There were 11 studies that reported on the effects of peer-led interventions on the behaviours of the peer leaders, though there were insufficient data to conduct a meta-analysis on these outcomes.

#### Narrative synthesis

A total of eleven studies reported on the behavioural outcomes of peer-leaders, five were quasi-experimental with a control group, and the remaining six were randomised controlled trials. The sample sizes of the included studies ranged from 42 to 945. Three of the studies reporting on behavioural outcomes ran for ten weeks or less, seven were more than ten weeks long, and the duration of one study could not be determined.

Whilst five studies reported at least one peer-led session per week, other studies did not focus on implementing formal sessions, instead encouraging leaders to promote certain behaviours through conversations with friends or by modelling these behaviours^9,10^. The remaining studies either did not report the number of peer-led sessions per week or had less than one session per week. In most cases where a study examined behavioural outcomes, peer-leaders either led a class or a small group of students (7/10), this information could not be found in one study. Regarding the age of the peer-leaders, there was a similar proportion of studies with children as peer-leaders (6/11) as there were with adolescents as leaders (5/11), and most utilised older students to lead younger students (7/11). Four of the included studies reported the use of a theory in the development of their intervention.

The outcomes of interest included physical activity, sexual activity and safety, dietary habits, and substance use. Some findings support that peer-led interventions may improve leaders’ behaviours; however, the evidence is mixed. Three studies examined the influence of peer-led sexual education interventions on the adolescent leaders’ sexual behaviours^44,50,51^. None had a statistically significant effect on the frequency of leaders’ sexual intercourse, though the intervention described by Caron, et al.^50^ had a significant positive effect on leaders’ use of condoms during sex, and Foss, et al.^51^ reported an improvement in leaders’ comfort in talking about sex and birth control to a date. Reporting on the findings of their peer-led drug education intervention, Golonka, et al.^52^ found that it reduced the increase in leaders’ alcohol consumption over time.

Of the six studies investigating physical activity as an outcome of interest, only one reported an increase in leaders’ physical activity^37^, though this was measured via self-report. Where leaders’ physical activity was assessed objectively (using pedometers or accelerometers), these interventions did not increase leaders’ physical activity^10,45^. Several studies examined the effect of their intervention on leaders’ self-reported diet and sedentary behaviours, though there was no evidence that school-based peer-led interventions reduce the sedentary behaviours of the leaders involved^53,54^. Finally, two studies found significant improvements in leaders’ self-reported diet, including a reduction in sugar-sweetened beverage consumption^9^ and an increased frequency of ‘heathy eating’^37^.

### Physical outcomes

#### Narrative synthesis

Only two studies examined the effect of peer-led interventions on physical markers of health in leaders (i.e., cardiorespiratory fitness, BMI, blood pressure, and waist circumference)^37,45^. Both implemented pilot studies for the ‘Healthy Buddies’ intervention, where a peer-leader delivered one lesson on healthy living to a younger student and participated in two physical activity sessions per week with them. The study described by Stock, et al.^37^ used a quasi-experimental design and included two schools, and Santos, et al.^45^ reported the findings from a larger randomised controlled trial. Both studies involved interventions that were implemented over the course of one school year. Combined, they included the results from 846 students. Neither study mentioned the use of a particular theory in the creation of the Healthy Buddies intervention. Despite improvements in several outcomes for the peer recipients, there were no significant physical health benefits to peer-leaders^37,45^.

## Discussion

The aim of our systematic review and meta-analysis was to examine the effect of school-based, peer-led interventions on leaders’ academic, behavioural, physical, and psychosocial outcomes. Our findings suggest that peer-led programs have large positive effects for leaders’ attitudes toward bullying (*d* = 1.02), small-to-medium positive effects for leaders’ literacy (*d* = 0.39), and small positive effects for leaders’ self-esteem (*d* = 0.18). There is some evidence that peer-led interventions may benefit certain behavioural outcomes of peer leaders, though overall, the findings were mixed. Further, there was no evidence that peer-led interventions benefit leaders’ physical outcomes, though only two studies examined these outcomes.

Across all of the included studies, the programs varied in their content, duration, dose, and in the age of the peer leaders. Unfortunately, there were not enough studies in each of our meta-analyses to examine the potentially moderating effects of these variations. Considering these variations, the pooled effect sizes represent the effect of participating in peer-led programs as a leader compared to age-matched controls not participating in such a program.

With regard to the small-to-medium effects of peer-led interventions on leaders’ literacy outcomes, the studies that informed this systematic review targeted a range of outcomes, including writing ability, reading comprehension, and vocabulary. Preparing to lead, as well as the act of leading itself, involves several processes that may provide some explanation for these effects. First, in preparation to lead, the leader must first revise the material and organise it into a format that they understand. The process of leading itself is a reciprocal one, whereby the peer recipient may ask questions or identify inconsistencies that require the leader to re-examine their own understanding of the content^55^. Given the evidence provided in this review and taking into consideration that these interventions also benefit the academic outcomes of peer recipients^13^, teachers may consider adopting peer-led interventions to improve students’ academic outcomes.

There are some key differences between the findings of the current and previous meta-analyses and reviews on the effect of peer-led programs on leaders’ academic outcomes. The current analysis did not include studies on populations with disabilities or behavioural issues, as has been done with other reviews^12,56^. The findings of the current review would appear to be more generalisable to typically developing young people. The mechanisms through which peer-led interventions improve leaders’ academic skills are unclear, and the effect of such interventions may be different for students with or without disabilities or behavioural issues. Therefore, the inclusion of these studies may conflate these effects. Previous reviews have also included studies utilising reciprocal teaching methods (where a student acts as the leader and the peer recipient)^13^. The inclusion of such studies precludes the isolation of the effect of being a leader in these interventions. Considering these factors, the evidence provided in the current analysis indicates that being a leader in peer-led interventions may be an effective means of improving academic outcomes in typically developing students.

Regarding the psychosocial outcomes, our findings suggest school-based, peer-led interventions have a small positive effect on leaders’ self-esteem. Similar to our findings, the authors of a recent systematic review of school-based peer-led interventions^5^ concluded that there are more documented benefits for leaders than there are for peer recipients for psychosocial outcomes. It is worth noting that in contrast to academic outcomes, psychosocial outcomes are not often the focus of these interventions. In their meta-analysis, Ginsburg-Block, et al.^57^ noted that peer-assisted learning focused on academic outcomes yielded similar effect sizes for psychosocial outcomes as interventions focused specifically on social skills. This is an important finding as it suggests focusing on academic outcomes may benefit psychosocial outcomes without targeting them. Indeed, changes in psychosocial outcomes may be driven by changes in leaders’ academic performance as a result of these interventions. For example, it is plausible that changes in self-esteem may result from changes in academic competence. Put another way, as a student becomes more proficient in their role as leader and in their content knowledge, their self-esteem may also benefit.

The three studies reported by Boulton, et al.^25^, reported on the effect of the ‘cross-age teaching zone (CATZ)’ anti-bullying intervention on leaders’ attitudes towards bullying. The authors’ hypothesised that by providing leaders with the opportunity to model content into a viable lesson, they would start to think about bullying in novel ways. Further, by delivering the content to younger students, the leaders may feel a sense of obligation to take their role seriously. Indeed, our meta-analysis of these studies indicate that the program has a large positive effect on leaders’ attitudes toward bullying. Whilst this provides some indication of the effectiveness of this program on this outcome, given that each study implemented the same program to similar populations, it is unclear how generalisable this result is to bullying programs utilised under different conditions. Accordingly, the large pooled effect on attitudes toward bullying is likely a better representation of the effect of the CATZ program, than of the effect of peer-led interventions more generally.

As children develop into adolescents, their peers become increasingly influential in shaping their health-related behaviours^58^. The involvement of students as advocates, leaders, and role-models may be an effective means of improving the health-related behaviours of those involved. Indeed, there is evidence to suggest that peer-led interventions are capable of improving the eating behaviours and physical activity of children and adolescents^8,59^. However, it remains unclear whether taking on the role of leader has an influence on their own behaviours. In the current review, not enough studies reported on leaders’ behavioural or physical outcomes to conduct corresponding meta-analyses. There was some evidence that peer-led interventions may influence leaders’ behaviours (including self-reported diet and alcohol consumption), although the findings were inconsistent. Modifying students’ behaviours is particularly complex, in part because it often involves actions that occur throughout a day, outside of the supervision of research staff and teachers^60^ (e.g., physical activity, diet, sleep, and sexual behaviour). This is further complicated by the fact that many students (particularly children) may have limited control over these behaviours. However, the effects of school-based peer-led interventions may differ according to whether a student is a leader, or a peer recipient. It may be the case that taking on the role and title of a leader serves as an incentive for the student to model certain behaviours, even away from the supervision of teachers and research staff. However, there is a need for more research on the behavioural outcomes of peer-leaders to establish whether behavioural effects differ according to the roles students adopt.

Only two studies examined whether their intervention influenced leaders’ physical outcomes^37,45^. Both studies implemented the ‘Healthy Buddies’ intervention, where peer leaders delivered lessons on healthy living and participated in physical activity sessions with their younger partner. In both studies, there were no increases in leaders’ physical activity, which may explain why there were no changes in BMI, blood pressure, or cardiorespiratory fitness. Given the limited number of studies that examined physical outcomes, it is inappropriate to draw conclusions on these outcomes at this time. Instead, we encourage future research to test the effect of peer-led interventions on peer-leaders’ behavioural and physiological outcomes. For instance, given the interest in peer-led physical activity interventions, we recommend that future research test their effects on leaders’ cardiorespiratory and muscular fitness. These tests are relatively easy to administer and provide a robust indicator of current and future health^61–63^.

There is a notable gap in the investigation of the effects of peer-led interventions on the leadership abilities of student leaders. Even where the development of students’ leadership ability is not the primary goal, the structure of a peer-led intervention necessitates that a student lead or guide others through a task or series of tasks. It is therefore plausible that, via the processes of teaching and leading other students, peer-leaders develop their own leadership abilities. Curiously, only two of the studies included in this review examined leadership outcomes^46,47^. The improvement in leadership skills over the course of an intervention may also provide some explanation for the outcomes of peer recipients, considering that as one’s leadership ability improves, so may their effectiveness in teaching or modelling a skill or behaviour. Accordingly, it is worth considering the inclusion of measures of leadership outcomes in future evaluations of peer-led interventions among youth.

Our assessment of the risk of bias of the included studies revealed that the biggest contributors to bias were the use of quasi-experimental designs and self-reported outcomes. These findings indicate that there is a need for more rigorous studies to be undertaken in this area of research. It is difficult to make recommendations on the use of self-report items, as for many constructs (including most psychosocial outcomes), self-report methods are the only method available (e.g., the assessment of self-concept or victimisation).

## Limitations

There are several limitations of this systematic review and meta-analysis that are worth noting. First, although we contacted authors, there were several studies that did not provide the information necessary to be included in the separate meta-analyses (n = 10). It is important that future research reports the information necessary for the computation of effect sizes so they may be included in future meta-analyses. Second, the high heterogeneity in the analyses of literacy and attitudes toward bullying suggests that other variables not assessed in this meta-analysis likely moderate the effect of peer-led interventions on these outcomes for leaders’. Some of this heterogeneity may be explained by the differences in the content, duration, dose, and in the age of the peer leaders. Unfortunately, our review included a relatively small number of studies, which limited our ability to conduct moderator analyses. Finally, we were unable to determine the extent to which the school-based, peer-led interventions were implemented as intended.

## Conclusion

This systematic review and meta-analysis provide evidence that school-based, peer-led interventions have positive effects on leaders’ academic and psychosocial outcomes. Our meta analyses indicate large positive effects on leaders’ attitudes toward bullying (*d* = 1.02), small-to-medium positive effects on leaders’ literacy (*d* = 0.39), and small positive effects on leaders’ self-esteem (*d* = 0.18). Evidence of the effect of these interventions on the health-related behaviours of leaders (including physical activity and diet) is limited and requires further investigation. There is a need for more research to determine the mechanisms responsible for these effects, and to identify the contribution of specific features of these interventions on leaders’ academic, behavioural, psychosocial, and physical outcomes.

## Supplementary Information

Supplementary Information 1.

Supplementary Information 2.

Supplementary Information 3.

## Data Availability

The data that support the findings of this study are available on request from the corresponding author, DRL.
