# Supplementary material for: A systematic review and meta-analysis of the benefits of school-based, peer-led interventions for leaders
Source: Sci Rep. 2022 Dec 8;12:21222. doi: 10.1038/s41598-022-25662-9 (PMC9732042; doi:10.1038/s41598-022-25662-9)
Supplement: Supplementary file 2 — Supplementary Information 2. [file 41598_2022_25662_MOESM2_ESM.docx]

## Appendix A - Search strategy (used medical sub-headings where appropriate)

## Population

Child* or adolescen* or youth* or teen* or student or “young people” or “young person”

AND

## Design

RCT or “randomized controlled trial” or “randomised controlled trial” or experiment* or quasi-experiment* or intervention

AND

## Intervention content

“peer-led* or leader* or tutor* or tutee or “peer-assisted learning” or mentor*

AND

## Location

School* or elementary* or “high school” or “primary school” or “middle school” or education*

Sportdiscus, search ran on the 24^th^ of October, 2022.

| **#** | **Query** | **Limiters/Expanders** | **Results** |
| --- | --- | --- | --- |
| S30 | S26 AND S27 AND S28 AND S29 | Expanders - Apply equivalent subjects Search modes - Boolean/Phrase | 457 |
| S29 | S20 OR S21 OR S22 OR S23 OR S24 OR S25 | Expanders - Apply equivalent subjects Search modes - Boolean/Phrase | 460066 |
| S28 | S14 OR S15 OR S16 OR S17 OR S18 OR S19 | Expanders - Apply equivalent subjects Search modes - Boolean/Phrase | 33626 |
| S27 | S8 OR S9 OR S10 OR S11 OR S12 OR S13 | Expanders - Apply equivalent subjects Search modes - Boolean/Phrase | 144325 |
| S26 | S1 OR S2 OR S3 OR S4 OR S5 OR S6 OR S7 | Expanders - Apply equivalent subjects Search modes - Boolean/Phrase | 261863 |
| S25 | education* | Expanders - Apply equivalent subjects Search modes - Boolean/Phrase | 277530 |
| S24 | "middle school" | Expanders - Apply equivalent subjects Search modes - Boolean/Phrase | 3444 |
| S23 | "primary school" | Expanders - Apply equivalent subjects Search modes - Boolean/Phrase | 2753 |
| S22 | "high school" | Expanders - Apply equivalent subjects Search modes - Boolean/Phrase | 29204 |
| S21 | elementary* | Expanders - Apply equivalent subjects Search modes - Boolean/Phrase | 11353 |
| S20 | school* | Expanders - Apply equivalent subjects Search modes - Boolean/Phrase | 263122 |
| S19 | mentor* | Expanders - Apply equivalent subjects Search modes - Boolean/Phrase | 4554 |
| S18 | "peer-assisted learning" | Expanders - Apply equivalent subjects Search modes - Boolean/Phrase | 37 |
| S17 | tutee | Expanders - Apply equivalent subjects Search modes - Boolean/Phrase | 10 |
| S16 | tutor* | Expanders - Apply equivalent subjects Search modes - Boolean/Phrase | 981 |
| S15 | "peer-led" | Expanders - Apply equivalent subjects Search modes - Boolean/Phrase | 146 |
| S14 | leader* | Expanders - Apply equivalent subjects Search modes - Boolean/Phrase | 28467 |
| S13 | intervention | Expanders - Apply equivalent subjects Search modes - Boolean/Phrase | 75852 |
| S12 | quasi-experiment* | Expanders - Apply equivalent subjects Search modes - Boolean/Phrase | 1752 |
| S11 | experiment* | Expanders - Apply equivalent subjects Search modes - Boolean/Phrase | 75128 |
| S10 | "randomised controlled trial" | Expanders - Apply equivalent subjects Search modes - Boolean/Phrase | 1529 |
| S9 | "randomized control trial" | Expanders - Apply equivalent subjects Search modes - Boolean/Phrase | 433 |
| S8 | RCT | Expanders - Apply equivalent subjects Search modes - Boolean/Phrase | 2835 |
| S7 | "young person" | Expanders - Apply equivalent subjects Search modes - Boolean/Phrase | 105 |
| S6 | "young people" | Expanders - Apply equivalent subjects Search modes - Boolean/Phrase | 5263 |
| S5 | student* | Expanders - Apply equivalent subjects Search modes - Boolean/Phrase | 90409 |
| S4 | teen* | Expanders - Apply equivalent subjects Search modes - Boolean/Phrase | 37543 |
| S3 | youth* | Expanders - Apply equivalent subjects Search modes - Boolean/Phrase | 36043 |
| S2 | adolescent* | Expanders - Apply equivalent subjects Search modes - Boolean/Phrase | 38174 |
| S1 | child* | Expanders - Apply equivalent subjects Search modes - Boolean/Phrase | 103495 |
