# Supplementary material for: A systematic review and meta-analysis of the benefits of school-based, peer-led interventions for leaders
Source: Sci Rep. 2022 Dec 8;12:21222. doi: 10.1038/s41598-022-25662-9 (PMC9732042; doi:10.1038/s41598-022-25662-9)

Appendix B - Risk of Bias of Included Studies

| **Author, year** | **1. Risk of Bias Judgement (Randomization)** | **2. Risk of Bias Judgement (Deviation)** | **3. Risk of Bias Judgement (Missing data)** | **4. Risk of Bias Judgement (Outcome Measurement)** | **5. Risk of Bias Judgement (Selection of Results)** | **Overall Risk of Bias Judgement** |
| --- | --- | --- | --- | --- | --- | --- |
| **Boulton 2016** | Some Concerns | Some Concerns | Some Concerns | Low Risk | Some Concerns | Some Concerns |
| **Boulton 2021** | Some Concerns | Some Concerns | Low Risk | Some Concerns | Some Concerns | Some Concerns |
| **Caron 2004** | High Risk | Some Concerns | High Risk | High Risk | Some Concerns | High Risk |
| **Campbell 2012** | High Risk | Some Concerns | Low Risk | Some Concerns | Some Concerns | High Risk |
| **Carruth 2010** | High Risk | Some Concerns | Some Concerns | Some Concerns | Some Concerns | High Risk |
| **Cui 2012** | Low Risk | Low Risk | Low Risk | High Risk | Some Concerns | High Risk |
| **Foss 2022** | Low Risk | Low Risk | Low Risk | High Risk | Low Risk | High Risk |
| **Golonka 2017** | High Risk | Some Concerns | Low Risk | High Risk | Some Concerns | High Risk |
| **Jago 2021** | Low Risk | Some Concerns | Low Risk | Some Concerns | Low Risk | Some Concerns |
| **Mason-Jones 2013** | High Risk | Some Concerns | High Risk | High Risk | Some Concerns | High Risk |
| **Miller 2010** | Low Risk | High Risk | Some Concerns | High Risk | Some Concerns | High Risk |
| **Mitchell 2016** | High Risk | High Risk | Low Risk | Some Concerns | Some Concerns | High Risk |
| **Nathan 2017** | High Risk | Some Concerns | Low Risk | High Risk | Some Concerns | High Risk |
| **Palladino 2012** | High Risk | Some Concerns | High Risk | High Risk | Some Concerns | High Risk |
| **Paquette 2009** | High Risk | High Risk | Low Risk | Some Concerns | Some Concerns | High Risk |
| **Robinson 2007** | High Risk | Some Concerns | Low Risk | High Risk | Some Concerns | High Risk |
| **Santos 2014** | Low Risk | Some Concerns | Low Risk | High Risk | Low Risk | High Risk |
| **Sheppard 2012** | High Risk | Some Concerns | High Risk | High Risk | Some Concerns | High Risk |
| **Silverman a 2017** | High Risk | Some Concerns | High Risk | Low Risk | Some Concerns | High Risk |
| **Silverman b 2017** | High Risk | Some Concerns | Low Risk | Low Risk | Some Concerns | High Risk |
| **Smit 2016** | Low Risk | High Risk | Some Concerns | High Risk | Low Risk | High Risk |
| **Song 2018** | High Risk | Some Concerns | High Risk | Low Risk | Some Concerns | High Risk |
| **Stock 2007** | High Risk | Some Concerns | High Risk | High Risk | Some Concerns | High Risk |
| **Tarro 2019** | Low Risk | Some Concerns | Low Risk | High Risk | Low Risk | High Risk |
| **Topping 2004** | High Risk | High Risk | High Risk | High Risk | Some Concerns | High Risk |
| **VanKeer 2010** | High Risk | Some Concerns | Some Concerns | High Risk | Some Concerns | High Risk |
| **Wong 2012** | Low Risk | Some Concerns | Low Risk | High Risk | Some Concerns | High Risk |
| **Wyman 2010** | Low Risk | Some Concerns | Low Risk | High Risk | Some Concerns | High Risk |
| **Yogev 1982** | High Risk | Some Concerns | Low Risk | High Risk | Some Concerns | High Risk |


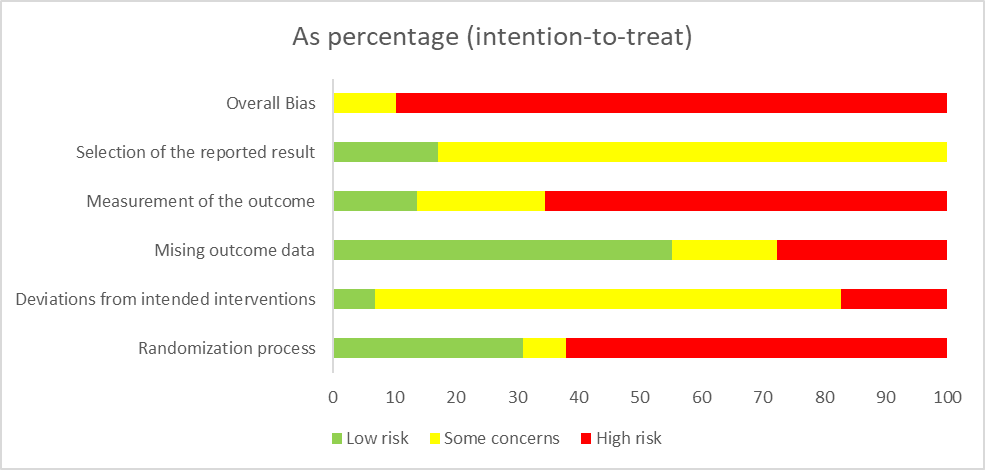

Supplement: Supplementary file 3 — Supplementary Information 3. [file 41598_2022_25662_MOESM3_ESM.docx]
